# Endings and beginnings

**Published:** 2016-03-28

**Authors:** W Ombelet

**Affiliations:** Genk Institute for Fertility Technology, ZOL Hospitals, Schiepse Bos 6, 3600 Genk, Belgium.

At the start of a new year it is time to look back over the past 12 months, as well as to look forward. 2015 has been one with a sad farewell to two very good friends, Dr Howard Jones Jr (see Fact Views Vis Obgyn, 2015, 7:149-152) and Professor Jan van der Merwe whose obituary appears in this issue.

The past year has seen some very interesting and stimulating papers, not to forget about the most original 2015 cover designed by Koen Vanmechelen.

The March issue was almost entirely dedicated to the second International Ovarian Tumour Analysis (IOTA) congress, held in Leuven, Belgium. We published eight outstanding papers linked to the different topics of the meeting and written by world experts in the field. I’d like to thank Professor Dirk Timmerman for helping us with the work-out and the content of this issue.

In the June issue special attention has been paid to sperm donors and the related problems caused to the offspring and the children’s family. The editorial written by Professor Guido Pennings reflected on the ongoing debate about sperm anonymity in Belgium.

In the September issue, Professor Wiebren Tjalma tackled the reimbursement for bone loss prevention in women and the discrepancies between reimbursement policies for women and men. Professor Geeta Nargund wrote a viewpoint paper describing the urgent need for fertility education in schools, a hot topic in the United Kingdom.

In the fourth issue of 2015 we noticed two very interesting reviews. In the first systematic review (Vandendriessche et al.) the authors examined the value of old and new promising techniques in directing more effective adjuvant therapy for breast cancer. The second review by Datta et al. showed us that many additional interventions used in assisted reproduction are being practiced with little justifiable evidence. They only make IVF treatment more expensive with questionable actual advantage.

In this issue we are very happy with an extremely interesting review on the religious aspects of assisted reproduction written by Professor Hassan Sallam from Alexandria, Egypt. I really believe that this paper should be a regarded as a one of the best reviews ever, describing the influence of religion on the human response to new developments such as assisted reproduction.

I sincerely hope that the future will remain exciting with a lot of interesting debates, original scientific work, strong opinions and unexpected viewpoints.

On behalf of the editorial board and the publisher I would like to thank all those who were involved in *Facts, Views & Vision in ObGyn* in 2015, our contributors, referees, secretarial team, the staff at Universa Press, our artistic reviewer and, of course, our readers ...


Willem Ombelet
Editor-in-Chief


